# The effect of body mass index and its interaction with family history on hypertension: a case–control study

**DOI:** 10.1186/s40885-019-0111-2

**Published:** 2019-02-21

**Authors:** An-le Li, Qian Peng, Yue-qin Shao, Xiang Fang, Yi-ying Zhang

**Affiliations:** Jiading district center for disease control and prevention, Shanghai, China

**Keywords:** Hypertension, Case-control study, Body mass index, Family history, Interaction

## Abstract

**Background:**

BMI is an indicator commonly used in the world to measure the weight and height of the body, it reflects the comprehensive outcome of acquired lifestyle; FH is a sign reflecting the main role of genetic factors. This study aimed to evaluate the effect of BMI and interaction with FH on hypertension risk in Shanghai adult population.

**Methods:**

According to l:l matched pairs design, 342 cases and 342 controls were selected and investigated in this study, this study was performed in Shanghai, China. Participants received face-to-face questionnaire survey, anthropometric tests and laboratory examinations. Relevant indicators that reflect obesity including BMI and waist to hip ratio (WHR) were calculated. Multivariate logistic regression analysis was applied to explore the association between factors and hypertension risk. Interactive effect was evaluated by synergy index (SI), relative excess risk due to interaction (RERI), attributable proportion due to interaction (AP) and the percentage of the interaction between the pure factors (PAP).

**Results:**

Among 684 study participants aged 28–87 years old, the differences of mean age and height between case group and control group are no significant (*p* > 0.05), but the differences of mean of weight, WC, HC, BMI and WHR are significant (*p* < 0.001). The OR of FH on hypertension is 4.986 (95%CI: 2.832~ 8.877); the OR of BMI on hypertension is respectively: low weight is 1.528 (95%CI: 0.551~ 4.239), overweight is 3.333 (95%CI: 1.678~ 6.617) and obesity is 7.312 (95%CI: 3.556~ 15.035). The OR of interaction between FH and BMI to hypertension is 12.993 (95%CI: 7.426~22.734). SI is 1.90 (95% CI: 1.48~3.78), RERI is 5.67 (95% CI: 1.66~11.88), AP is 43.87% (95% CI: 12.84~91.88%), and PAP is 47.55% (95%CI: 13.91~99.58%). FH and BMI have positive interaction on hypertension. 43.87% of hypertension exposed to both FH and BMI was attributable to the interaction of them.

**Conclusions:**

FH and BMI are significant higher risks of hypertension; with the increase of BMI, the risk of hypertension will increase more. FH and BMI have positive interaction with hypertension, the interaction is greater than the sum of two independent actions.

## Introduction

Some epidemiological studies showed that the prevalence of hypertension has significantly increased among children and adolescents in recent years, and hypertension have affected 20 to 30% of the population worldwide [1–3]. As is known to all, hypertension is a multifactorial disease caused by genetic and environmental factors, the role of gene and gene, as well as between gene and environment, leads to increased risk of hypertension and disease among different populations. Unhealthy lifestyle such as obesity and lack of exercise can significantly raise hypertension [4, 5]. The result of familial aggregation of hypertension showed that in positive population of parents, the prevalence rate of brothers and sisters in offspring is as high as 20 to 66%, a plurality of twin studies have estimated the possibility of hereditary is over 50% [6]. It shows that more than half of the blood pressure change can be attributed to the accumulation of genetic effects.

It is assumed that blood pressure is controlled by a large number of genes, and each gene has only a relatively weak effect on blood pressure. Therefore, it is difficult to detect genetic variants that affect blood pressure by traditional methods such as candidate gene screening and gene linkage studies. Family history (FH) is an important marker of genetic factors, it is often used as an alternative indicator to study the relationship between genetic factors and diseases [7–10]. Body mass index (BMI) is a comprehensive indicator of the outcome of acquired lifestyle, and closely related to the occurrence of hypertension [11–15]. A review of meta-analytic studies has shown that general obesity is measured by BMI, central and abdominal obesity is measured by anthropometric indictor such as waist circumference or waist-to-hip ratio, and obesity is associated with a risk of hypertension and cardiovascular disease mortality [16].

The aim of this study is to evaluate the effect of BMI and interaction with family history on hypertension risk in Shanghai adult population.

## Methods

### Study population

This case–control study included aged 28 to 87 years old; all participants gave informed consent to participate in the study which was approved by the local ethics committee. All cases are randomly selected from hypertension registry and follow-up management system, they are confirmed by hospital and verified by follow-up in the community, they were able to correctly respond to the investigators for health information of themselves. The inclusion criteria of the cases are that it meets the following three conditions at the same time: 1) diagnosed as essential hypertension patients and had been verified by the community management; 2) residents of the district or Shanghai household registration; 3) formally signed the informed consent and voluntarily participated. And the exclusion criteria of the cases are that it meets any of the following conditions: 1) patients with secondary hypertension cannot be excluded; 2) patients with renal insufficiency or psychosis; 3) patients with poor compliance.

According to l: l matched pairs design, all controls had no hypertension, and gave informed consent to participate in the study, they were able to correctly respond to the investigators for the health information of themselves. The inclusion criteria of the controls are that it meets the following three conditions at the same time: 1) no blood relationship with the case; 2) the same sex and race; 3) the same place of residence; 4) the same age or age difference less than 5 years old.

Family history refers to at least one or more patients with essential hypertension among their lineal relatives, requiring all patients to be distributed within three generations.

## Measurements

### Anthropometric measurements

Waist circumference (WC) was measured at a level midway between the lower rib margin and the iliac crest. Hip circumference (HC) was measured at the maximum circumference around the buttocks. WC and HC were measured with using a flexible measuring tape, the accuracy ±0.5 cm. Height of the participants was measured with portable height measurer, the accuracy ±0.1 cm; and weight was measured with SECA measuring equipment (wearing only light clothing and barefooted), the accuracy ±0.1 kg.

Body mass index (BMI) = body weight (kg) / height squared(m^2^). BMI classification standard: BMI < 18.5 is low weight (thin); BMI between 18.5~23.9 is normal weight; BMI between 24.0~27.9 is overweight; BMI over 28.0 is obesity [17, 18]. The participants were grouped into the following categories of BMI: low weight (thin), normal weight, overweight and obese. WHR ≤0.90 (male) and WHR ≤ 0.80 (female) are normal; WHR > 0.90 (male) and WHR > 0.80 (female) are abnormal.

### Statistical analysis

Statistical analyses were performed using the statistical software package (IBM SPSS statistics version 21). When *P* values < 0.05, the difference was considered statistically significant. Mean and standard deviation (SD) were used to compute for quantitative variables (age, weight, height, WC, HC, BMI and WHR), and comparisons between groups were performed by t-test. Number (n) and percentage (%)) were computed for the categorical data, comparisons between groups were performed by the chi-square (*χ*^2^) test. Univariate and multivariate logistic regression analyses were conducted for investigated risk factors, odds ratios (OR) and 95% confidence intervals (CI) were calculated. In multivariate analysis, OR were adjusted by sex.

The additive model was used by cross analysis to calculate the additive interaction effect. The synergistic effect index (SI), relative excess risk due to interaction (RERI), attributable proportion due to interaction (AP) and the percentage of the interaction between the pure factor (PAP) calculation formula are as follows [19]:

$$ {\displaystyle \begin{array}{l} SI(AB)=\left[R(AB)-R\left({A}^0{B}^0\right)\right]/\left[\ R\left({AB}^0\right)-R\left({A}^0{B}^0\right)+R\left({A}^0B\right)-R\left({A}^0{B}^0\right)\right]\\ {} RERI(AB)=\left[\ R(AB)-R\left({AB}^0\right)-R\left({A}^0B\right)+R\left({A}^0{B}^0\right]/R\right({A}^0{B}^0\\ {} AP(AB)=\left[R(AB)-R\left({AB}^0\right)-R\left({A}^0B\right)+R\left({A}^0{B}^0\right)\right]/R(AB)\\ {} PAP(AB)=\left[\ R(AB)-R\left({AB}^0\right)-R\left({A}^0B\right)+R\left({A}^0{B}^0\right)\right]/\left[R(AB)-R\left({A}^0{B}^0\right)\right]\end{array}} $$

*(Note: R(AB) is the risk ratio of A and B factor exposed; R(A* ^*0*^ *B* ^*0*^ *) is the risk ratio of A and B factor unexposed; R(AB* ^*0*^ *) is the risk ratio of A factor exposed but B factor unexposed, R(A* ^*0*^ *B) is the risk ratio of A factor unexposed and B factor exposed)*

## Results

### Distribution of participants

In this study, 342 hypertension cases and 342 control populations were investigated. Table 1 shows the distribution of measurement data of the study population in two groups. Among 684 participants aged 28–87 years old, the difference of mean age and height between case group and control group are no significant (*p* > 0.05), but the difference of mean Weight, WC, HC, BMI and WHR between case group and control group are significant (*p* < 0.001). The mean of BMI, WHR, weight, WC and HC in case group are significantly higher than that of control group. See Table 1.

**Table 1:** Distribution of measurement variables in participants in two groups

	control group (*n* = 342)		case group (*n* = 342)		t	p
	mean	SD	mean	SD		
Age (years)	61.69	10.73	62.42	10.72	0.894	0.372
BMI (kg/m^2^)	23.46	3.18	25.52	3.50	8.032	<0.001
WHR	0.90	0.06	0.92	0.06	4.392	<0.001
Height (cm)	163.30	8.10	162.81	7.94	0.799	0.424
Weight (kg)	62.62	10.04	67.75	11.35	6.262	<0.001
WC (cm)	83.73	9.96	88.69	9.09	6.806	<0.001
HC (cm)	92.79	9.26	96.07	8.22	4.898	<0.001

*BMI* body mass index, *WHR* waist-to-hip ratio, *WC* waist circumference, *HC* hip circumference

Table 2 shows the distribution of categorical data of participant in the study. Among these study participants, 76.17% participants have family history of hypertension (FH), 23.83% have not FH. 43.42% participants are normal weight, 2.49% are low weight, 40.64% overweight and 13.45% obesity. 81.43% of WHR are abnormal. Sex distribution: 50.73% male; 49.27% female. Education level: 30.56% primary school and below; 61.84% high school; 7.60% college and above. Blood type: 61.99% knew and 38.01% unknown. Occupation: 68.28% engaged; 6.29% freelance and 25.44% no job. Work or life pressure: 90.79% no feeling or feel little, 9.21% feel more. Living environmental noise: 91.81% no feeling or feel little, 8.19% feel more. Personal taste: 22.37% like salty taste and 31.29% like light taste. Sleeping time: 11.40% feel inadequate; 88.60% feel adequate. Sports habit: 18.86% never exercise and 81.14% exercise. Drinking behavior: 71.20% never drink and 28.80% drink. Smoking behavior: 64.91% never smoking and 35.09% smoking.

**Table 2:** Distribution of categorical variables in participants in two groups (N, %)

		control group (*n* = 342)		case group (*n* = 342)			
		n	%	n	%	x^2^	p
sex	male	175	51.17	172	50.29	0.119	0.730
	female	167	48.83	170	49.71		
Education level	Below primary	20	5.85	22	6.43	6.567	0.161
	primary school	84	24.56	83	24.27		
	junior high	135	39.47	152	44.44		
	high school	69	20.18	67	19.59		
	≥college	34	9.94	18	5.26		
family history	no	131	38.30	32	9.36	80.048	<0.001
	yes	211	61.70	310	90.64		
body mass index	low weight	12	3.51	5	1.46	63.165	<0.001
	normal	194	56.73	103	30.12		
	overweight	112	32.75	166	48.54		
	obesity	24	7.02	68	19.88		
waist-to-hip ratio	normal	74	21.64	53	15.50	4.492	0.034
	abnormal	268	78.36	289	84.50		
blood type	AB	41	11.99	33	9.65	3.523	0.620
	A	52	15.20	57	16.67		
	B	49	14.33	55	16.08		
	O	65	19.01	72	21.05		
	unknown	135	39.47	125	36.55		
occupation	physical labor	154	45.03	152	44.44	12.197	0.016
	mental work	90	26.32	71	20.76		
	freelance	26	7.60	17	4.97		
	no job	72	21.05	102	29.82		
work/life pressure	no	217	63.45	222	64.91	3.08	0.544
	little	96	28.07	85	24.85		
	more	23	6.73	24	7.02		
	great	6	1.75	10	2.92		
Living environmental noise	no	231	67.54	223	65.20	3.22	0.521
	little	85	24.85	89	26.02		
	more	21	6.14	27	7.89		
	great	5	1.46	3	0.88		
Personal taste	salty	65	19.01	88	25.73	5.738	0.057
	light	104	30.41	110	32.16		
	balance	172	50.29	146	42.69		
sleeping time	inadequate	43	12.57	35	10.23	6.998	0.072
	satisfy	31	9.06	49	14.33		
	sufficient	268	78.36	258	75.44		
Sports habit	never	64	18.71	65	19.01	1.454	0.483
	occasionally	116	33.92	129	37.72		
	regular	162	47.37	148	43.27		
drinking behavior	never	236	69.01	251	73.39	2.068	0.558
	occasionally	65	19.01	59	17.25		
	often	38	11.11	33	9.65		
smoking behavior	never	225	65.79	219	64.04	6.544	0.087
	Quit now	22	6.43	40	11.70		
	occasionally	20	5.85	24	7.02		
	always	72	21.05	61	17.84		

Between case group and control group, the statistical test results shows that the difference of sex, education, blood type, work and life pressure, living environmental noise, person’s taste, sleeping time, sports habit, drinking behavior and smoking behavior is no significant (*p* > 0.05). But the difference of family history (FH), BMI, WHR and occupation between case group and control group is significant (*p* < 0.05).

### Effect of risk factors on hypertension

According to the logistic regression analysis models, after adjustment for sex, the result shows that effect of BMI,WHR, FH and drinking behavior are significant (*p* < 0.05). But effect of age, education, blood type, occupation, work and life pressure, environmental noise, taste, sleeping time, sports habit and smoking behavior are not significant (*p* > 0.05). See Table 3.

**Table 3:** The logistic regression analysis results of investigated risk factors on hypertension

Variables	B	S.E,	Wals	df	Sig.	OR	OR 95% C.I.	
							lower	upper
Age	−0.255	0.205	1.551	1	0.213	0.775	0.519	1.157
Education	0.091	0.103	0.791	1	0.374	1.096	0.896	1.339
BMI	−0.843	0.143	34.893	1	< 0.001	1.430	1.325	1.569
WHR	−0.561	0.247	5.148	1	0.023	1.571	1.352	1.926
Blood type	−0.006	0.066	0.008	1	0.927	0.994	0.874	1.131
Occupation	−0.096	0.078	1.502	1	0.220	0.909	0.780	1.059
work/life pressure	0.156	0.155	1.023	1	0.312	1.169	0.864	1.583
environmental noise	−0.057	0.145	0.154	1	0.695	0.945	0.711	1.255
Taste	0.228	0.121	3.558	1	0.059	1.256	0.991	1.593
Sleeping time	−0.260	0.142	3.344	1	0.067	0.771	0.584	1.019
Sports	0.090	0.125	0.512	1	0.474	1.094	0.856	1.398
Drinking	0.330	0.163	4.093	1	0.048	1.391	1.010	1.914
Smoking	−0.080	0.09	0.788	1	0.375	0.924	0.775	1.101
Family history	1.412	0.221	40.733	1	< 0.001	4.103	2.660	6.33
constant	2.234	1.119	3.986	1	0.046	9.334		

*BMI* body mass index, *WHR* waist-to-hip ratio

BMI is an indicator commonly used in the world to measure the weight and height of the body, it reflect the comprehensive outcome of acquired lifestyle; family history is an important marker of genetic factors effect. In order to further evaluate the effect and interaction on hypertension, risk factors of FH and BMI are selected on the next analysis. The interclass correlation coefficient was 0.248 (*p* < 0.001), the results showed that the reliability coefficient was statistically significant. Multilevel logistic regression analysis is used to analyze the individual effect of FH and BMI on hypertension, the result shows in Table 4.

**Table 4:** The multilevel logistic regression analysis results of FH and BMI on hypertension

Variables		B	S.E,	Wals	df	Sig.	OR	OR 95% C.I.	
								lower	upper
FH	Yes	1.425	0.205	48.234	1	<0.001	4.986	2.832	8.877
BMI				63.098	3	<0.001			
	<18.5	0.424	0.521	6.620	1	0.046	1.528	0.551	4.239
	24.0~27.9	1.204	0.291	17.092	1	<0.001	3.333	1.679	6.617
	≥28.0	1.989	0.368	29.260	1	<0.001	7.312	3.556	15.035

*FH* family history of hypertension, *BMI* body mass index

Table 4 shows the individual effect of FH and BMI to hypertension by multilevel logistic regression model analysis, and all results are statistical significant. The OR between FH and hypertension is 4.986 (95%CI: 2.832~ 8.877); the OR between low weight (BMI < 18.5) and hypertension is 1.528 (95%CI: 0.551~ 4.239), the OR between overweight (BMI 24.0~27.9) and hypertension is 3.333 (95%CI: 1.679~ 6.617), the OR between obesity (BMI ≥ 28.0) and hypertension is 7.312 (95%CI: 3.556~ 15.035).

### Interaction of FH and BMI

Table 5 shows the result of interactive effects analysis. The OR of interaction between FH and BMI to hypertension is 12.993 (95%CI: 7.426~22.734). OR_FH + BMI_ > OR_FH_ + OR_BMI_. It is showed that FH and BMI have positive interaction with hypertension. According the result of Table 5, use additive model to calculate the additive interaction effect: the synergistic effect index (SI) of FH and BMI to hypertension is 1.90 (95% CI: 1.48~3.78), relative excess risk due to interaction (RERI) is 5.67 (95% CI: 1.66~11.88), attributable proportion due to interaction (AP) is 43.87% (95% CI: 12.84~91.88%), and the percentage of the interaction between the pure factor (PAP) is 47.55% (95%CI: 13.91~99.58%). The result of AP indicates that 43.87% of hypertension was attributable to the interaction of them, when exposed to both FH and BMI risk factors.

**Table 5:** The interaction between FH and BMI to hypertension

FH	BMI	case group	control group	OR	95%CI
–	–	20	75	1.000	
–	+	32	36	3.333	1.679~6.617
+	–	117	88	4.986	2.832~8.777
+	+	246	70	12.993	7.426~22.734

[FH: “-” no, “+” yes; BMI: “-” normal weight, “+” abnormal weight (include thin, overweight and obese)]

## Discussion

Previous studies have shown that hypertension has obvious familial clustering and the family history of hypertension has a heritability of 60%, more than a half of the objects in these studies have family history of hypertension. Compared with patients without family history of hypertension, patients with a family history of hypertension have a lower onset age and higher blood pressure levels, and it indicating that genetic factors can lead to elevated blood pressure levels and advanced onset age [20, 21]. FH of hypertension is an important marker of genetic factors. In this study, there are 76.17% of participants have family history of hypertension, and the effect of FH are significant between case group and control group; The OR of FH on hypertension is 4.986 (95%CI: 2.832~ 8.877), it is clearly showed that FH is an important risk factor of hypertension.

As BMI is a weight-for-height measure, it does not distinguish between fat mass and lean mass [22]. A cross-sectional study in the United States reported a significantly higher risk for elevated BP in the participants with high BMI [23]; In another study, BMI was significantly associated with an increased risk of prehypertension [24]; The current study showed the importance of the interactions of different anthropometric indicators of obesity in assessing the risk of hypertension; overweight/obesity can assess cardiovascular risk in children and adolescents [25, 26]. Obesity is a risk factor for the development of hypertension, which can increase hypertension through multiple mechanisms, including insulin resistance, activation of sympathetic nervous system, sodium retention leading to increased renal reabsorption and activation of the renin–angiotensin system [27–30]. The increasing populations of overweight and obese residents suggest the potential risk of increasing incidence of hypertension [31]. The population of overweight and obesity may even be under- estimated because the standard definitions for overweight and obesity used in research may be too high for Asian population [32, 33]. Moreover, abdominal obesity can be present in individuals with normal BMI values (18.5–24.9 kg/m^2^), and some studies have indicated that this condition could be a risk factor for hypertension [34, 35]. Due to the small relatively size of human bodies in Asia, fat usually tends to accumulate in the abdomen, forming central obesity, and central obesity can lead to chronic non-communicable diseases [36–39].

This study shows that the difference of mean weight and BMI between case group and control group is significant (*p* < 0.001), the mean of BMI and weight in case group are significantly higher than that of control group. The difference of BMI effect between two groups is significant (*p* < 0.05), the OR of low weight (BMI < 18.5) is 1.528, OR of overweight (BMI 18.5~23.9) is 3.333, OR of obesity (BMI ≥ 24.0) is 7.312. The OR of interaction between FH and BMI to hypertension is 12.993 (95%CI: 7.426~22.734). OR_FH + BMI_ > OR_FH_ + OR_BMI_, the interaction is greater than the sum of two independent actions, it is showed that FH and BMI have positive interaction with hypertension.

There are many factors affecting the incidence of hypertension, such as hereditary factors and acquired factors. There are many ways of interactions among these factors. Synergistic effect index (SI), relative excess risk due to interaction (RERI), attributable proportion due to interaction (AP) and the percentage of the interaction between the pure factor (PAP) were used to quantitatively measure interactions [19, 40, 41]. SI can be used for quantitative and qualitative analysis of interaction. In this study, we quantified interaction by additive model, SI is 1.90 (> 1), it shows that FH and BMI have positive interaction with hypertension. RERI is used for quantitative analysis of interaction, this study shows RERI is 5.67, it shows that interaction between FH and BMI is 5.67. AP is used to calculate the proportion attributable to interactions after background effects are removed. This study shows AP is 43.87%, it shows that attributable proportion due to interaction between FH and BMI is 43.87%. PAP can explain the degree of harm of exposure factors to a population, and the extent to which these factors may reduce the incidence of disease after elimination, that is the social effects of exposure. This study shows PAP is 47.55%, it shows that the percentage of the interaction between FH and BMI is 47.55%. Owing to genetic factors are the factors that cannot be changed, but overweight and obesity are modifiable risk factors, effective proactive intervention programme could help slow and ultimately reduce the number of individuals with obesity becoming hypertensive.

Our study has some limitations. First, the current study explored only by case- control study, and the number of samples is limited. Therefore, our findings need to be confirmed and extended in further larger population and cohort study. The second, in the current study, there was no detailed analysis for WHR, education, blood type, occupation, work and life pressure, environmental noise, taste, sleeping time, sports habit and smoking behavior, for considering that these factors classification are not very elaborate, and it is also not the focus of this study.

## Conclusions

FH and BMI are significant higher risk for hypertension, the risk of hypertension will increase more with the increase of BMI; FH and BMI have positive interaction with hypertension, the interaction is greater than the sum of two independent actions.

## Abbreviations

BMI: Body mass index
CI: confidence interval
FH: family history of hypertension
HC: Hip circumference
OR: odds ratio
WC: Waist circumference
WHR: Waist-to-height ratio
